# The relationship between striatal dopamine and anterior cingulate glutamate in first episode psychosis changes with antipsychotic treatment

**DOI:** 10.1038/s41398-023-02479-2

**Published:** 2023-05-31

**Authors:** Sameer Jauhar, Robert A. McCutcheon, Mattia Veronese, Faith Borgan, Matthew Nour, Maria Rogdaki, Fiona Pepper, James M. Stone, Alice Egerton, George Vamvakas, Federico Turkheimer, Philip K. McGuire, Oliver D. Howes

**Affiliations:** 1 Psychological Medicine, Institute of Psychiatry, Psychology and Neuroscience, King’s College, London, UK; 2 Department of Psychiatry, University of Oxford, Oxford, UK; 3 Oxford Health NHS Foundation Trust, Oxford, UK; 4 Department of Psychosis Studies, Institute of Psychiatry, Psychology and Neuroscience, King’s College, London, UK; 5 Centre for Neuroimaging Sciences, Institute of Psychiatry, Psychology and Neuroscience, King’s College, London, UK; 6 Max Planck UCL Centre for Computational Psychiatry and Ageing Research, Berlin, Germany; 7 Department of Neuroscience and Imaging, University of Sussex, Brighton and Hove, UK; 8 Department of Biostatistics, Institute of Psychiatry, Psychology and Neuroscience, King’s College, London, UK; 9 MRC London Institute of Medical Sciences, Imperial College, London, UK; 10 H Lundbeck A/s, St Albans, UK

**Keywords:** Neuroscience, Schizophrenia

## Abstract

The neuromodulator dopamine and excitatory neurotransmitter glutamate have both been implicated in the pathogenesis of psychosis, and dopamine antagonists remain the predominant treatment for psychotic disorders. To date no study has measured the effect of antipsychotics on both of these indices together, in the same population of people with psychosis. Striatal dopamine synthesis capacity (Ki^cer^) and anterior cingulate glutamate were measured using 18F-DOPA positron emission tomography and proton magnetic resonance spectroscopy respectively, before and after at least 5 weeks’ naturalistic antipsychotic treatment in people with first episode psychosis (*n* = 18) and matched healthy controls (*n* = 20). The relationship between both measures at baseline and follow-up, and the change in this relationship was analyzed using a mixed linear model. Neither anterior cingulate glutamate concentrations (*p* = 0.75) nor striatal Ki^cer^ (*p* = 0.79) showed significant change following antipsychotic treatment. The change in relationship between whole striatal Ki^cer^ and anterior cingulate glutamate, however, was statistically significant (*p* = 0.017). This was reflected in a significant difference in relationship between both measures for patients and controls at baseline (*t* = 2.1, *p* = 0.04), that was not present at follow-up (t = 0.06, p = 0.96). Although we did not find any effect of antipsychotic treatment on absolute measures of dopamine synthesis capacity and anterior cingulate glutamate, the relationship between anterior cingluate glutamate and striatal dopamine synthesis capacity did change, suggesting that antipsychotic treatment affects the relationship between glutamate and dopamine.

## Introduction

The dopamine hypothesis of psychosis remains one of the predominant biological theories within psychiatry [1–4], and one central strand is the clinical efficacy of dopamine D_2_ antagonists [5–8]. Dopamine interactions with other neurotransmitter systems have been implicated in psychosis, these including GABA [9], serotonin [10] and the endocannabinoid system [11], though the majority of literature has examined the excitatory neurotransmitter, glutamate [12].

Pre-clinical models show interactions between the dopamine and glutamate systems which could contribute to the actions of antipsychotics [12]. Microdialysis experiments show dopamine antagonists cause an acute increase in extracellular dopamine, which reverts to baseline levels upon chronic treatment [13]. Rodent spectroscopy suggests effects of antipsychotics on frontal cortex glutamate, with olanzapine and clozapine decreasing this, though no change was seen with aripiprazole, haloperidol or risperidone [14]. Moreover, drug challenge studies have demonstrated targeting one system may have reciprocal effects, for example, acute ketamine increasing cortical, striatal and nucleus accumbens dopamine in-vivo [15].

Striatal dopamine synthesis capacity (Ki^cer^) can be measured in-vivo using positron emission tomography, and cortical glutamate can be measured using proton magnetic resonance spectroscopy (MRS). Effects on separate components of the dopamine and glutamate systems have been examined in few in-vivo studies [16] but not together in the same population. One study showed a decrease in Ki^cer^ with sub-chronic haloperidol in 9 people with schizophrenia free of antipsychotic medication [17], whilst another found no difference in whole striatal Ki^cer^ in 17 people with first episode psychosis, initially not taking antipsychotic medication, who were then treated naturalistically with second generation antipsychotics [18]. A systematic review of in-vivo MRS studies found a small decrease in Glx (glutamate + glutamine) in some brain regions following antipsychotic treatment [19], another study showing reduction in anterior cingulate cortex (ACC) glutamate in people with first episode schizophrenia [20]. Conversely, a recent study in 61 people with schizophrenia failed to show any change in ACC or hippocampal glutamate after 6 weeks' risperidone treatment [21].

In-vivo examination of both systems in the same population has been limited to two cross-sectional studies. A study in healthy volunteers reported a direct relationship between medial prefrontal cortex glutamate and striatal dopamine synthesis capacity [22]. While in people with first episode psychosis, we reported an inverse correlation between ACC glutamate and striatal Ki^cer^ [23]. We are unaware of studies examining effects of antipsychotics on both systems in the same people, which is necessary if one wishes to examine interactions between both systems.

In the current study we obtained measures of both anterior cingulate glutamate concentrations, and striatal dopamine synthesis capacity, before and after treatment with antipsychotics, in the same cohort of individuals with first episode psychosis. We hypothesized no overall within-subject change in Ki^cer^ or glutamate, instead predicting that antipsychotics would produce more subtle circuit-level changes in both neurotransmitters, reflected in a change in the relationship between the two measure pre- and post-treatment.

## Methods and materials

Ethical approval was given by East of England-Cambridge East Ethics Committee, and the Administration of Radioactive Substances Advisory Committee (ARSAC). All participants provided informed written consent. The patient group had scans at baseline and after antipsychotic treatment, the healthy control sample having scans solely at baseline.

### Participants

Patients (*N* = 18) were recruited from London first episode psychosis (FEP) services and were required to be experiencing their first episode of psychotic illness, antipsychotic naïve, free of antipsychotics for >6 weeks or minimally treated with antipsychotics for <2 weeks. For inclusion, subjects required a diagnosis of a psychotic disorder meeting International Classification of Disease-10 (ICD 10) criteria [24], and experience psychotic symptoms, defined as at least moderate severity on one or more of the delusion (P1), hallucination (P3), and persecution (P6) items on the Positive and Negative Syndrome Scale (PANSS), consistent with previous studies [24, 25]. Diagnosis was confirmed by a study psychiatrist (SJ), using a structured instrument (Mini-international Neuropsychiatric Interview (MINI)) [26]. Inclusion criteria required people with psychosis to be antipsychotic naïve, antipsychotic-free for at least 6 weeks, or “minimally treated” (receiving antipsychotic medication for 2 weeks or less).

Healthy control subjects (*N* = 20) were recruited from the same geographical area as the patient group. Inclusion criteria for controls were: no personal history of psychiatric illness (assessed using the MINI) and no concurrent psychotropic medication (through self-report).

Exclusion criteria for all participants were: history of significant head trauma (any loss of consciousness due to head injury), dependence on illicit substances (defined using the MINI), medical co-morbidity (other than minor illnesses), family history of psychosis and contra-indications to scanning (such as pregnancy). Nicotine and alcohol use were permitted, though specific restrictions were placed on the day of PET.

### Antipsychotic treatment

All antipsychotic doses were required to be within therapeutic range, defined in the Maudsley Prescribing Guidelines [27]. Use of other psychotropic medication (such as antidepressants and benzodiazepines) was permitted. To assess concordance we used a multisource approach, requiring evidence of adequate adherence on at least two of the following: antipsychotic plasma levels, pharmacy and electronic medical records, and self-report from the patient and an independent source (family member/caregiver or health care professional). Adequate concordance was defined as taking a minimum of 80% of prescribed doses, in line with consensus recommendations [28]. To measure antipsychotic exposure, we determined chlorpromazine-equivalent dose years, as described by Andreasen et al. [29]. In the cases of lurasidone and amisulpride, we used the method described by Leucht et al. [30], using data from the Maudsley Prescribing Guidelines [27], because these are not covered in Andreasen et al.

### Clinical measures

Symptoms were measured using the positive and negative syndrome scale (PANSS), with raters blinded to imaging results. Age, gender and ethnicity (white/non-white) were also recorded.

### PET imaging acquisition and analysis

All participants were asked not to eat or drink (except water), and refrain from alcohol for 12 h prior to scan. Imaging data were obtained on a Siemens Biograph 6 HiRez PET scanner (Siemens, Erlangen, Germany) in three-dimensional mode. One hour before scan, participants received 400 mg entacapone, a peripheral catechol-*o*-methyl-transferase inhibitor to prevent formation of radiolabeled metabolites that may cross the blood–brain barrier, and 150 mg carbidopa, a peripheral aromatic acid decarboxylase inhibitor to increase the PET imaging signal. Participants were positioned in the scanner with the orbitomeatal line parallel to the transaxial plane of the tomograph. Head position was marked, monitored and movement minimized using a head strap. After acquiring a CT scan for attenuation correction, ~150 MBq ^18^F-DOPA was administered by bolus intravenous injection, 30 s after the start of PET imaging. PET data were acquired in 32 frames of increasing duration over the 95-min scan (frame intervals: 8 × 15 s, 3 × 60 s, 5 × 120 s, 16 × 300 s).

Correction for head movement during scan was performed by employing a mutual information algorithm, described in prior publications [30, 31]. SPM 8 [31, 32] was used to automatically normalize a tracer-specific ^18^F-DOPA template [32, 33] together with the striatal brain atlas as defined by Martinez et al. [33, 34]. The primary outcome measure, the striatal influx constant for whole striatum with cerebellum as the reference region, Ki^cer^ (1/min), was calculated using the Patlak-Gjedde graphical approach adapted for a reference tissue input function, used in prior studies by our group [24, 25, 30, 31, 34, 35].

### MRS acquisition

All scans were acquired on a General Electric (Milwaukee, Wisconsin, USA) Signa

HDxt 3 Tesla MRI scanner. Internal localizer scans were used to determine the anterior commissure-posterior commissure line and inter-hemispheric angle. For the voxel placements, 3D coronal inversion recovery prepared spoiled gradient echo (IR-SPGR) scans were acquired, followed by auto pre-scans for optimization of water suppression and shimming. A T1 weighted structural scan was also obtained and was used for subsequent segmentation and CSF correction. ^1^H-MRS spectra were acquired for the anterior cingulate region-of-interest (right-left 20 mm × anterior-posterior 20 mm x superior-inferior 20 mm). The anterior cingulate cortex voxel was prescribed from the midline sagittal localizer, with the centre of the 20 × 20 × 20 mm voxel placed 13 mm above the genu of corpus callosum perpendicular to the AC–PC line to minimize inclusion of white matter and cerebral spinal fluid (CSF). ^1^H-MRS spectra (Point RESolves Spectroscopy (PRESS), TE = 30 ms, TR = 2 s) were obtained through the PROton Brain Examination (PROBE) sequence by GE, which includes water suppression.

### MRS analysis

Water-scaled metabolites, using a standard basis set of 16 metabolites (L-alanine, aspartate, creatine, phosphocreatine, GABA, glucose, Gln, glutamate, glycerophosphocholine, glycine, myo-inositol, L-lactate, N-acetylaspartate, N-acetylaspartylglutamate, phosphocholine, and taurine), provided with LCModel and generated using same field strength (3 Tesla), localization sequence (PRESS), and echo time (30 msec)/ The acquired data were analyzed using LC-model 6.3-I0 [36] and we specifically estimated levels of glutamate, in keeping with our previous study, which highlighted the relationship between whole striatal ^18^F-DOPA PET and anterior cingulate Glutamate, in people with first episode psychosis [23].

Spectra were visually inspected and metabolite analyses were restricted to spectra with line width (full-width at half-maximum; FWHM) ≤ 0.1 ppm, Cramér-Rao lower bounds (CRLB) for glutamate ≤ 20%, signal to noise ratio ≥ 5. Corrections were applied to account for relative distribution of cerebrospinal fluid within anterior cingulate. In-house scripts were used to identify relative distribution of white, grey matter and cerebrospinal fluid in the voxel prescribed to the anterior cingulate. The following correction was subsequently applied in order to correct for CSF content within the voxel; where M raw metabolite value, WM white matter and GM grey matter:

$${{{\mathrm{Mcorr}}}} = {{{\mathrm{M}}}} \ast ({{{\mathrm{WM}}}} + {{{\mathrm{GM}}}} \ast 1.22 + {{{\mathrm{CSF}}}} \ast 1.55)/({{{\mathrm{WM}}}} + {{{\mathrm{GM}}}})$$

### Statistical analysis

Analyses were performed using Stata version 13 [37] and R version 3.3.2 [38]. Linear mixed effect models were constructed to determine effects of treatment on dopamine synthesis capacity, glutamate concentration, and the relationship between them.

Our primary analysis investigated whether the association between striatal Ki^cer^ and anterior cingulate glutamate observed in the patient group at baseline changed following antipsychotic treatment. In this analysis Ki^cer^ was the dependent variable. Glutamate, timepoint (baseline vs follow-up), and a glutamate * time point interaction were included as fixed effects, with a random participant-level effect. The effect of treatment on glutamate and dopamine individually was examined with a linear mixed model in which the neurochemical measure in question was the dependent variable, time point was a fixed effect, with a random participant-level effect.

Secondary analyses investigated whether striatal Ki^cer^ or anterior cingulate glutamate changed individually. In these analyses the neurochemical measure was the dependent variable, while time was included as a fixed effect, with a random participant-level effect. In addition dopamine-glutamate association in patients was compared with the association observed in controls, by fitting a linear model with Ki^cer^ as the dependent variable, and glutamate, group and glutamate* group interaction as independent variables. This model was fitted separately for baseline and follow-up scans for the patient group

## Results

### Study participants

20 healthy controls received baseline scans, while 18 people with first episode psychosis (FEP) received both baseline and follow-up scans and clinical assessment. Demographic details are given in Table 1. There were no significant differences between patients and controls in age, gender or ethnicity.

**Table 1:** Demographic details.

	Controls (*N* = 20)	Patients(*N* = 18)		*P*-value
**Age**	23.5 (±3.4)	25.0 (±3.2)		0.17
**Sex (%male)**	13 (65.0%)	14 (77.8%)		0.61
**Ethnicity (% white)**	13 (70%)	8 (44%)		0.34
**Medication status**	N/A			
**Antipsychotic Naive**		10		
**Antipsychotic Free**		5		
**Minimally Treated**		3 (risperidone 2mg, *n* = 1; amisulpride 200mg, *n* = 1, amisulpride 300mg *n* = 1)		
		Baseline	Followup	*P*-value
**PANSS Positive**		20.3(±6.7)	12.9 (±5.8)	<0.001
**PANSS Negative**		15.8(±5.0)	12.4 (±5.9)	0.04
**PANSS General**		37.1(±8.1)	27.6 (±9.7)	<0.001
**PANSS Total**		73.8(±16.0)	52.9 (±19.6)	0.001
**Ki**^**cer**^ *10^**-3**^ **(min**^**-1**^**)**	12.9 (±1.2)	12.9(±1.1)	12.9 (±1.0)	0.55
**Acc Glu (Institutional Units)**	14.1 (±2.1)	13.7(2.2)	13.8 (±1.6)	0.75
**Median time between PET and MRS at baseline**	4 days (IQR = 8)			
**Median time between PET and MRS at follow-up**		6 days (IQR = 18)		
**Time between** ^**18**^**F-DOPA scans**		68 days (IQR = 46)		
**Time between MRS scans**		77 days (IQR = 111)		

There was no statistical difference between groups, in terms of illicit drug use (self-report) or urine drug screen, nicotine or alcohl use (self-report).

At baseline the mean total PANSS in the patient group was 73.8 (SD 16.0) which reduced at a statistically significant level (*p* < 0.01) to 52.9 (SD 19.6) following antipsychotic treatment.

Time between PET and MRS was as follows. Baseline; median 3.5 days (IQR = 5.75)

Follow-up; median 5.5 days (IQR 17.5 days).

At baseline, ten patients were antipsychotic naïve, five were medication free and three were minimally treated. All patients received a minimum of 4 weeks’ antipsychotic treatment between baseline and follow-up scans.

### Psychotropic medication

One patient was using Sertraline at baseline, and one taking benzodiazepines at follow-up.

Psychotropic medication during the study is given in Table 2.

**Table 2:** Psychotropic medication.

Psychotropic medication
Amisulpride, *N* = 7
Amisulpride + Aripiprazole *N* = 1*N* = 1
Amisulpride, Quetiapine *N* = 1
Amisulpride, Olanzapine *N* = 1
Aripiprazole, *N* = 1
Lurasidone *N* = 1
Olanzapine, *N* = 1
Paliperidone *N* = 1
Quetiapine, *N* = 1
Quetiapine + Sertraline, *N* = 1
Risperidone, Aripiprazole, *N* = 1
Risperidone, Sodium Valproate *N* = 1
Risperidone, *N* = 1

The median chlorpromazine dose years of antipsychotic treatment was 0.32 (IQR 0.17).

### Change in dopamine and glutamate measures

**Fig. 1:**
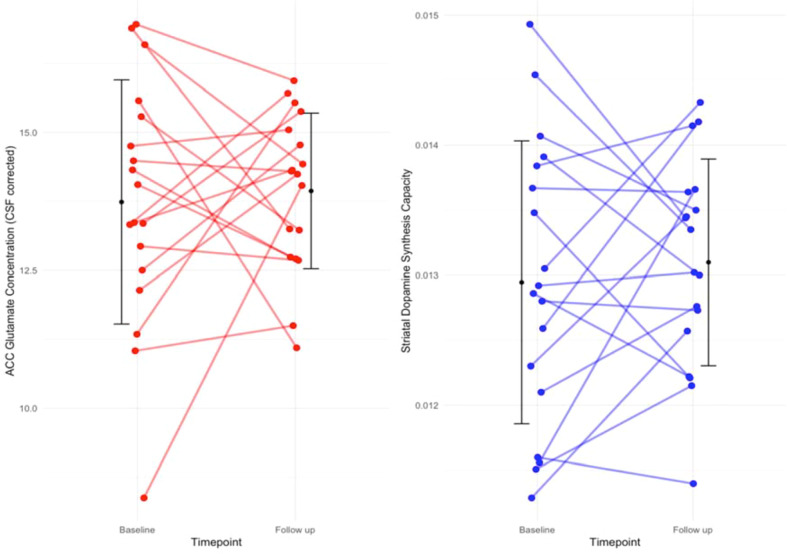
Change in individual dopamine and glutamate measures. Neither anterior cingulate glutamate concentrations (*p* = 0.53) nor striatal ^18^F-DOPA Ki^cer^ (*p* = 0.74) showed a significant change following antipsychotic treatment.

As reported in a sub-sample of these patients [18], there was no significant change in whole striatal Ki^cer^ with antipsychotic treatment (coefficient = 1.5*10^−4^, SE = 2.4*10^−4^, *p* = 0.53) (see Fig. 1). There was no significant change in anterior cingulate glutamate concentrations (coefficient = 0.20, SE = 0.60, *p* = 0.74) (see Fig. 1). MRS quality metrics and checklist are given in Tables 3 and 4.

**Table 3:** MRS Quality Metrics.

	Baseline Mean	Baseline SD	Follow-up Mean	Follow-up SD	Control Mean	Control SD	*P*-values
**Spectra quality measures**							
**FWHM**	0.03938889	0.007204075	0.03729412	0.007703609	0.03885	0.006968161	0.6792491
**SN**	19.27777778	4.944462804	19.41176471	4.258210063	18.10000	5.035662294	0.6503201
**CRLB**	7.16666667	1.917412472	6.94117647	1.144038255	7.45000	1.848897253	0.6579231
Segmentation measures							
**CSF**	0.2263711	0.04606015	0.2312759	0.04356772	0.22975	0.03986540	0.9418366
**GM**	0.6640037	0.04044277	0.6604720	0.03603106	0.67015	0.02926517	0.7000233
**WM**	0.1094820	0.03422825	0.1080831	0.03267300	0.10000	0.02981346	0.6190083

FWHM Full Width Half Maximum

SN Signal; noise ratio

CRLB Cramér Rao Lower Bounds

CSF cerebrospinal fluid

The *p*-value reflects the results of an ANOVA between the three sets of measurements.

**Table 4:** MRS checklist.

MRS checklist
**a. Field strength [T]**
3T
**b. Manufacturer**
General Electric
**c. Model (software version if available)**
GE Signa HDx running software version 14.0_M5_0737.f
**d. RF coils: nuclei (transmit/ receive), number of channels, type, body part**
8-channel receive only head coil by Invivo
**e. Additional hardware**
None
**2. Acquisition**
**a. Pulse sequence**
PRESS
**b. Volume of Interest (VOI) locations**
Anterior cingulate cortex
**c. Nominal VOI size [cm**^**3**^**, mm**^**3**^**]**
20 × 20 × 20 mm
**d. Repetition time (TR), Echo Time (TE) [ms, s]**
TR = 3000 ms;
TE = 30 ms
**e. Total number of excitations or acquisitions per spectrum**
96, with an additional 16 with water suppression off
In time series for kinetic studies; not relevant
Number of Averaged spectra (NA) per time-point
Averaging method (e.g. block-wise or moving average)
Total number of spectra (acquired / in time-series)
128 averages
**f. Additional sequence parameters**
(spectral width in Hz, number of spectral points, frequency offsets)
If STEAM:, Mixing Time (TM)
If MRSI: 2D or 3D, FOV in all directions, matrix size, acceleration factors, sampling method
PRESS, TR = 3000 ms, TE = 30 ms, #points = 4096, spectral width = 5 kHz, frequency offset of PRESS pulses = -2 ppm (ie applied at 2.7 ppm)
**g. Water suppression method**
CHESS
**h. Shimming method, reference peak, and thresholds for “acceptance of shim” chosen**
automated linear shimming using B0 maps.
**i. Triggering or motion correction method**
(respiratory, peripheral, cardiac triggering, incl. device used and delays)
None
**3. Data analysis methods and outputs**
**a. Analysis software**
LCModel version 6.3–10
**b. Processing steps deviating from quoted reference or product**
None
**c. Output measure**
(e.g. absolute concentration, institutional units, ratio) Processing steps deviating from quoted reference or product
Metabolite concentration in institutional units, corrected for voxel tissue fractions
**d. Quantification references and assumptions, fitting model assumptions**
LCModel basis set
**4. Data quality**
**a. Reported variables**
(SNR, Linewidth (with reference peaks)) See Table 3.
SNR ≥ 5
Spectra were visually inspected and metabolite analyses were restricted to spectra with line width (full-width at half-maximum; FWHM) ≤ 0.1 ppm
**b. Data exclusion criteria**
Metabolite CRLB > 20%
Cramér-Rao lower bounds (CRLB) for glutamate > 20%, signal to noise ratio < 5.
**c. Quality measures of postprocessing Model fitting (eg. CRLB, goodness of fit, SD of residual)**
CRLB See Supplementary Material
**d. Sample spectrum**
See Fig. 1, Supplementary Material

**Fig. 2:**
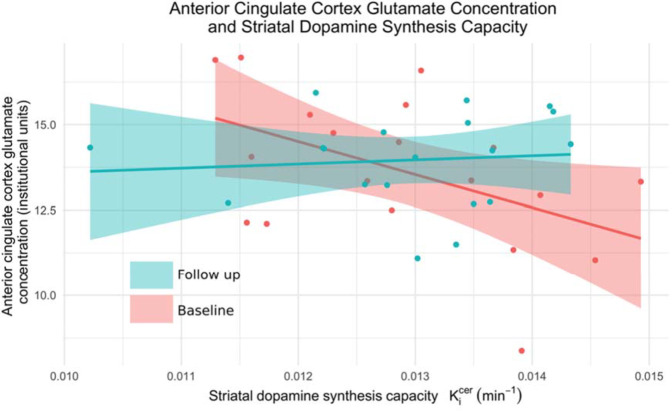
Dopamine-glutamate associations change following treatment. The relationship between ^18^F-DOPA Ki^cer^ and glutamate in patients at baseline (red) is significantly different from that observed following antipsychotic treatment (blue) (*p* = 0.02).

**Fig. 3:**
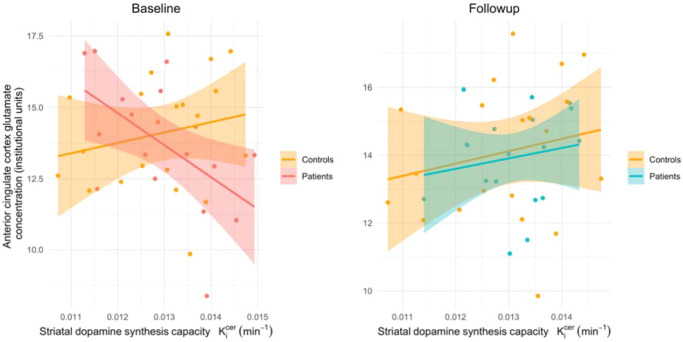
Dopamine-glutamate associations in patients before and after antipsychotic treatment compared to controls. The relationship between striatal ^18^F-DOPA K^cer^ and anterior cingulate glutamate in patients is significantly different from that observed in controls prior at baseline (*p* = 0.03). Following treatment there is no longer a significant difference between patients and controls (*p* = 0.93).

There was a significant interaction between glutamate and time (coefficient = 3.7*10^−4^, SE = 1.5*10^−4^, *p* = 0.018), reflecting a negative association between Ki^cer^ and ACC glutamate at baseline, that was not present at follow-up (see Fig. 2). This finding is also illustrated by the fact that at baseline there was a significant interaction between patients and controls in the dopamine-glutamate relationship (estimate = −3.0*10^−4^, SE = 1.7*10^−4^, *p* = 0.03, previously reported [23]). In contrast, where data obtained in patients at follow-up was compared to the same data obtained at the single timepoint in controls there was no difference between patients and controls in this dopamine-glutamate relationship (estimate = −1.7*10^−5^, SE = 2.1*10^−4^, *p* = 0.93, see Fig. 3). The interaction between Glx and time was not significant (coefficient = 1.8*10^−4^, SE = 1.0*10^−4^, *p* = 0.077).

## Discussion

We observed normalization of the relationship between striatal Ki_cer_ and anterior cingulate glutamate in people with first episode psychosis following antipsychotic treatment. This change in relationship was significant, and the follow-up dopamine-glutamate relationship was similar to that observed in healthy controls at baseline.

To the best of our knowledge this is the first study to examine relationships between striatal ^18^F-DOPA Ki^cer^ and anterior cingulate glutamate before and after antipsychotic treatment. Strengths of the study include the fact that the population under study consisted of patients with a first episode psychosis, and were predominantly antipsychotic free or naïve at baseline. Limitations include a modest sample size and naturalistic antipsychotic treatment. However, all antipsychotics were prescribed at valid treatment dose, and changes seen in PANSS indicated adequate clinical response in the majority of patients. The lack of placebo group means it is impossible to infer symptom change being wholly due to antipsychotic treatment,. A further weakness is the lack of follow-up data in the control group. Prior studies have examined changes in each of these measures separately in similar populations (first episode psychosis/schizophrenia) [17–19, 21], though both measures have not been examined together in the same population. Prior studies show conflicting results, with decreases in anterior cingulate glutamate following antipsychotic treatment suggested in a systematic review of small studies [19] and a relatively large sample (*n* = 46) [20], though no change in a relatively large study (*n* = 45) [21]. A decrease was seen in ^18^F-DOPA Ki in a sample of 9 people treated with sub-chronic haloperidol [17], though no change in a larger sample examined by our group, a subsample of which was examined here [18].

It is important to acknowledge the test/re-test reliability of both imaging measures. Regarding ^18^F-DOPA PET, inter-rater reliability was measured in 8 healthy controls, with an interclass correlation of 0.843 for Whole Striatum, and mean time between scans was (Mean ± SD 113.6 + /-16 weeks). The reliability of MRS Glu at 3 T has been measured in posterior cingulate cortex (PCC), using PRESS sequence, in 18 individuals (range 1 day–1 week), ICC = 0.8 [39].

As acknowledged [23], the glutamate signal at 3 T includes a contribution from Glutamnine (10–15%), and there is an inability to differentiate intracellular and extracellular glutamate concentrations. Similarly, our measure of dopamine synthesis capacity, aromatic acid decarboxylase (AADC) is not the rate-limiting enyme for dopamine synthesis, though remains the best tracer available, in terms of reliability and validity [25].

By applying CRLB threshold (>20%) as opposed to an absolute threshold, across all subjects, it is conceivable that if concentrations are lower in one group or time point, this group would have higher CRLBs [40]. However, we found no significant difference in concentrations at different time points, and therefore this effect is unlikely.

By examining the relationship between striatal ^18^F-DOPA Ki^cer^ and anterior cingulate glutamate, we suggest antipsychotic medication may exert effects on the relationship between these two measures. Specifically, the change in relationship is towards that seen in controls.

One model of psychosis pathoetiology proposes that dysregulation of cortical glutamatergic neurons [4, 41], through impaired GABA-ergic inhibition, leads to disinhibition of excitatory projections to dopamine neuron cell bodies in the midbrain, to stimulate dopamine neuron firing [41]. There is meta-analytic evidence that antipsychotics may reduce cortical glutamate levels in-vivo, in people with schizophrenia [42], although measures used, Magnetic Resonance Spectroscopy (MRS), are of total tissue glutamate rather than synaptic glutamate, it remains unclear to what degree this reflect glutamatergic neuronal activity. Notwithstanding, this could account for an uncoupling of the relationship between cortical glutamate and subcortical dopamine seen in our sample. However, it should be recognized that the current study does not show causality, and it remains possible that other effects underlie the alterations we report. In-vivo studies utilizing pharmacological manipulation of cortical glutamatergic activity are needed to disentangle these possibilities [43], as well as pre-clinical models. It should also be recognized that substance misuse, an aetiological factor in psychosis [44], may have similar effects on these systems, including the effects of cannabis use, decreasing cortical glutamate, seen in an MRS study of people with early psychosis [45]

### Future directions

This study requires replication in a larger sample, ideally with a control group scanned at both time points. Focusing on specific patient populations, eg those with lower (relative) dopamine synthesis capacity, may help delineate the interaction with cortical glutamate better, alongside better field strength MRS measures (7 T). It will also be of value to see how the association highlighted in the current study relates to other interaction effects observed using multimodal imaging [46–49], and any identified circuits could be further examined using pre-clinical models.

## Conclusions

We demonstrated a change in the relationship between measures of striatal dopamine synthesis capacity and anterior cingulate glutamate in first episode psychosis after antipsychotic treatment, the subsequent relationship being comparable to that seen in healthy controls.

## Supplementary information

SupplSupplementary Material

## References

[1] van Rossum JM (1966). The significance of dopamine-receptor blockade for the mechanism of action of neuroleptic drugs. Arch Int Pharmacodyn Thér.

[2] Meltzer HY, Stahl SM (1976). The dopamine hypothesis of schizophrenia: a review. Schizophr Bull.

[3] Howes OD, Kapur S (2009). The dopamine hypothesis of schizophrenia: version III-the final common pathway. Schizophr Bull.

[4] McCutcheon RA, Krystal JH, Howes OD (2020). Dopamine and glutamate in schizophrenia: biology, symptoms and treatment. World Psychiatry.

[5] Seeman P, Lee T (1975). Antipsychotic drugs: direct correlation between clinical potency and presynaptic action on dopamine neurons. Science.

[6] Creese I, Burt DR, Snyder SH (1976). Dopamine receptor binding predicts clinical and pharmacological potencies of antischizophrenic drugs. Science.

[7] Farde L, Nordström AL, Wiesel FA, Pauli S, Halldin C, Sedvall G (1992). Positron emission tomographic analysis of central D1 and D2 dopamine receptor occupancy in patients treated with classical neuroleptics and clozapine. Relation to extrapyramidal side effects. Arch Gen Psychiatry.

[8] Kapur S (2000). Relationship between dopamine D2 occupancy, clinical response, and side effects: a double-blind PET study of first-episode Schizophrenia. Am J Psychiatry.

[9] Garbutt JC, van Kammen DP (1983). The interaction between GABA and dopamine: implications for schizophrenia. Schizophr Bull.

[10] Kapur S, Remington G (1996). Serotonin-dopamine interaction and its relevance to schizophrenia. Am J Psychiatry.

[11] Covey DP, Mateo Y, Sulzer D, Cheer JF, Lovinger DM (2017). Endocannabinoid modulation of dopamine neurotransmission. Neuropharmacology.

[12] Carr DB, Sesack SR (2000). Projections from the rat prefrontal cortex to the ventral tegmental area: target specificity in the synaptic associations with mesoaccumbens and mesocortical neurons. J Neurosci.

[13] Samaha A-N, Seeman P, Stewart J, Rajabi H, Kapur S (2007). ‘Breakthrough’ dopamine supersensitivity during ongoing antipsychotic treatment leads to treatment failure over time. J Neurosci J Soc Neurosci.

[14] McLoughlin GA, Ma D, Tsang TM, Jones DNC, Cilia J, Hill MD (2009). Analyzing the effects of psychotropic drugs on metabolite profiles in rat brain using 1H NMR spectroscopy. J Proteome Res.

[15] Kokkinou M, Ashok AH, Howes OD (2018). The effects of ketamine on dopaminergic function: meta-analysis and review of the implications for neuropsychiatric disorders. Mol Psychiatry.

[16] Rogeau A, Nordio G, Veronese M, Brown K, Nour MM, Osugo M (2022). The relationship between glutamate, dopamine, and cortical gray matter: a simultaneous PET-MR study. Mol Psychiatry.

[17] Gründer G, Vernaleken I, Müller MJ, Davids E, Heydari N, Buchholz HG (2003). Subchronic haloperidol downregulates dopamine synthesis capacity in the brain of schizophrenic patients in vivo. Neuropsychopharmacology.

[18] Jauhar S, Veronese M, Nour MM, Rogdaki M, Hathway P, Natesan S (2019). The effects of antipsychotic treatment on presynaptic dopamine synthesis capacity in first-episode Psychosis: a positron emission tomography study. Biol Psychiatry.

[19] Egerton A, Bhachu A, Merritt K, McQueen G, Szulc A, McGuire P (2017). Effects of antipsychotic administration on brain glutamate in schizophrenia: a systematic review of longitudinal (1)H-MRS studies. Front Psychiatry.

[20] Egerton A, Broberg BV, Van Haren N, Merritt K, Barker GJ, Lythgoe DJ (2018). Response to initial antipsychotic treatment in first episode psychosis is related to anterior cingulate glutamate levels: a multicentre 1H-MRS study (OPTiMiSE). Mol Psychiatry.

[21] Kraguljac NV, Morgan CJ, Reid MA, White DM, Jindal RD, Sivaraman S, et al. A longitudinal magnetic resonance spectroscopy study investigating effects of risperidone in the anterior cingulate cortex and hippocampus in schizophrenia. Schizophr Res. 2019. 10.1016/j.schres.2018.12.028. 10.1016/j.schres.2018.12.028 PMC7881837 30630705

[22] Gleich T, Deserno L, Lorenz RC, Boehme R, Pankow A, Buchert R (2015). Prefrontal and striatal glutamate differently relate to striatal dopamine: potential regulatory mechanisms of striatal presynaptic dopamine function?. J Neurosci.

[23] Jauhar S, McCutcheon R, Borgan F, Veronese M, Nour M, Pepper F (2018). The relationship between cortical glutamate and striatal dopamine in first-episode psychosis: a cross-sectional multimodal PET and magnetic resonance spectroscopy imaging study. Lancet Psychiatry.

[24] Organization WH. The ICD-10 classification of mental and behavioural disorders: clinical descriptions and diagnostic guidelines. World Health Organization; 1992.

[25] Jauhar S, Nour MM, Veronese M, Rogdaki M, Bonoldi I, Azis M (2017). A test of the transdiagnostic dopamine hypothesis of psychosis using positron emission tomographic imaging in bipolar affective disorder and Schizophrenia. JAMA Psychiatry.

[26] Sheehan DV, Lecrubier Y, Sheehan KH, Amorim P, Janavs J, Weiller E (1998). The Mini-International Neuropsychiatric Interview (MINI): the development and validation of a structured diagnostic psychiatric interview for DSM-IV and ICD-10. J Clin Psychiatry.

[27] Taylor DM, Barnes TRE, Young AH. The Maudsley prescribing guidelines in psychiatry. 13th edn. Hoboken, NJ: Wiley-Blackwell; 2018.

[28] Howes OD, McCutcheon R, Agid O, de Bartolomeis A, van Beveren NJM, Birnbaum ML (2016). Treatment-resistant Schizophrenia: Treatment Response and Resistance in Psychosis (TRRIP) working group consensus guidelines on diagnosis and terminology. Am J Psychiatry.

[29] Andreasen NC, Pressler M, Nopoulos P, Miller D, Ho B-C (2010). Antipsychotic dose equivalents and dose-years: a standardized method for comparing exposure to different drugs. Biol Psychiatry.

[30] Leucht S, Samara M, Heres S, Patel MX, Woods SW, Davis JM (2014). Dose equivalents for second-generation antipsychotics: the minimum effective dose method. Schizophr Bull.

[31] Jauhar S, Veronese M, Rogdaki M, Bloomfield M, Natesan S, Turkheimer F (2017). Regulation of dopaminergic function: an [18F]-DOPA PET apomorphine challenge study in humans. Transl Psychiatry.

[32] SPM. Statistical parametric mapping. 2016. http://www.fil.ion.ucl.ac.uk/spm/.

[33] Egerton A, Mehta MA, Montgomery AJ, Lappin JM, Howes OD, Reeves SJ (2009). The dopaminergic basis of human behaviors: a review of molecular imaging studies. Neurosci Biobehav Rev.

[34] Martinez D, Narendran R, Foltin RW, Slifstein M, Hwang D-R, Broft A (2007). Amphetamine-induced dopamine release: markedly blunted in cocaine dependence and predictive of the choice to self-administer cocaine. Am J Psychiatry.

[35] Veronese M, Santangelo B, Jauhar S, D'Ambrosio E, Demjaha A, Salimbeni H (2021). A potential biomarker for treatment stratification in psychosis: evaluation of an [18 F] FDOPA PET imaging approach. Neuropsychopharmacology.

[36] LCModel’s home page. http://s-provencher.com/pages/lcmodel.shtml. Accessed 12 September 2016.

[37] StataCorp. Stata statistical software: release 13. College Station, TX: StataCorp LP; 2013.

[38] The R Foundation. R: The R project for statistical computing. https://www.r-project.org/. Accessed 24 February 2018.

[39] Baeshen A, Wyss PO, Henning A, O’Gorman RL, Piccirelli M, Kollias S (2020). Test–retest reliability of the Brain Metabolites GABA and Glx With JPRESS, PRESS, and MEGA-PRESS MRS Sequences in vivo at 3T. J Magn Reson Imaging.

[40] Kreis R (2016). The trouble with quality filtering based on relative Cramér-Rao lower bounds. Magn Reson Med.

[41] McCutcheon RA, Reis Marques T, Howes OD (2020). Schizophrenia—An overview. JAMA Psychiatry.

[42] Merritt K, McGuire PK, Egerton A, 1H-MRS in Schizophrenia Investigators, Aleman A, Block W, et al. Association of age, antipsychotic medication, and symptom severity in Schizophrenia with proton magnetic resonance spectroscopy brain glutamate level: a mega-analysis of individual participant-level data. JAMA Psychiatry. 2021. 10.1001/jamapsychiatry.2021.0380. 10.1001/jamapsychiatry.2021.0380 PMC8060889 33881460

[43] Pillinger T, Rogdaki M, McCutcheon RA, Hathway P, Egerton A, Howes OD (2019). Altered glutamatergic response and functional connectivity in treatment resistant schizophrenia: the effect of riluzole and therapeutic implications. Psychopharmacol (Berl).

[44] Jauhar S, Johnstone M, McKenna PJ (2022). Schizophrenia. Lancet Lond Engl.

[45] Rigucci S, Xin L, Klauser P, Baumann PS, Alameda L, Cleusix M (2018). Cannabis use in early psychosis is associated with reduced glutamate levels in the prefrontal cortex. Psychopharmacol (Berl).

[46] McCutcheon RA, Marques TR, Howes OD (2019). Schizophrenia—An overview. JAMA Psychiatry.

[47] McCutcheon RA, Nour MM, Dahoun T, Jauhar S, Pepper F, Expert P (2019). Mesolimbic dopamine function is related to salience network connectivity: an integrative positron emission tomography and magnetic resonance study. Biol Psychiatry.

[48] Limongi R, Jeon P, Mackinley M, Das T, Dempster K, Théberge J (2020). Glutamate and dysconnection in the salience network: neurochemical, effective-connectivity, and computational evidence in schizophrenia. Biol Psychiatry.

[49] McCutcheon RA, Pillinger T, Rogdaki M, Bustillo J, Howes OD (2021). Glutamate connectivity associations converge upon the salience network in schizophrenia and healthy controls. Transl Psychiatry.

