# Supplementary figures and images for: The relationship between striatal dopamine and anterior cingulate glutamate in first episode psychosis changes with antipsychotic treatment
Source: Transl Psychiatry. 2023 May 31;13:184. doi: 10.1038/s41398-023-02479-2 (PMC10229638; doi:10.1038/s41398-023-02479-2)

**Supplementary Ma**Figure 1

Sample Spectra

**
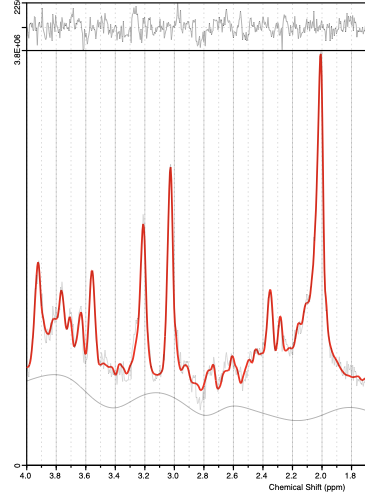
**

Supplement: Supplementary file 1 — SupplSupplementary Material [file 41398_2023_2479_MOESM1_ESM.docx]
